# Disruption of the brain-derived neurotrophic factor (BDNF) immunoreactivity in the human Kölliker-Fuse nucleus in victims of unexplained fetal and infant death

**DOI:** 10.3389/fnhum.2014.00648

**Published:** 2014-09-04

**Authors:** Anna M. Lavezzi, Melissa F. Corna, Luigi Matturri

**Affiliations:** “Lino Rossi” Research Center for the Study and Prevention of Unexpected Perinatal Death and SIDS Department of Biomedical, Surgical and Dental Sciences, University of MilanMilan, Italy

**Keywords:** human brainstem development, Kölliker-Fuse nucleus, BDNF, SIDS, SIUDS, gliosis, apoptosis, maternal smoking in pregnancy

## Abstract

Experimental studies have demonstrated that the neurotrophin brain-derived neutrophic factor (BDNF) is required for the appropriate development of the central respiratory network, a neuronal complex in the brainstem of vital importance to sustaining life. The pontine Kölliker-Fuse nucleus (KFN) is a fundamental component of this circuitry with strong implications in the pre- and postnatal breathing control. This study provides detailed account for the cytoarchitecture, the physiology and the BDNF behavior of the human KFN in perinatal age. We applied immunohistochemistry in formalin-fixed and paraffin-embedded brainstem samples (from 45 fetuses and newborns died of both known and unknown causes), to analyze BDNF, gliosis and apoptosis patterns of manifestation. The KFN showed clear signs of developmental immaturity, prevalently associated to BDNF altered expression, in high percentages of sudden intrauterine unexplained death syndrome (SIUDS) and sudden infant death syndrome (SIDS) victims. Our results indicate that BDNF pathway dysfunctions can derange the normal KFN development so preventing the breathing control in the sudden perinatal death. The data presented here are also relevant to a better understanding of how the BDNF expression in the KFN can be involved in several human respiratory pathologies such as the Rett's and the congenital central hypoventilation syndromes.

## Introduction

To accomplish all vital homeostatic functions, a proper respiratory rhythmogenesis is crucial at birth. The central ventilatory rhythm is produced and coordinated by a network of neuronal centers located in the brainstem (Ramirez and Richter, 1996; Viemari et al., 2003; Wong-Riley and Liu, 2005). Experimental studies have demonstrated that during prenatal development this circuitry undergoes marked maturational changes which include modifications in the morphological, biochemical and electrophysiological properties of specific neurons of the respiratory components (Ritter and Zhang, 2000; Feldman et al., 2003; Alheid and McCrimmon, 2008), as well as enhancement of their synaptic interactions (Paton and Richter, 1995), so that it will be fully developed and operating at birth.

The pontine Kölliker-Fuse (KF) is a key nucleus in the respiratory network for primary breathing, with strong implications in pre- and postnatal life. During intrauterine life, the KF inhibits the central and peripheral chemoreceptors (which are already fully developed and potentially functional), and therefore any respiratory reflex. At birth, the KF abruptly reduces its inhibitory effects and becomes active as a respiratory center that starts up the ventilatory activity through extensive afferent and efferent connections with the other respiratory-related structures (Dutschmann et al., 2004; Ezure, 2004; Dutschmann and Herbert, 2006).

Studies by Erickson et al. (1996) firstly demonstrated that brain-derived neurotrophic factor (BDNF), a member of the neurotrophin class of growth factors, is required for the development of normal breathing in mice, both for regulating the maturation of specific neurons in brainstem centers involved in respiratory control, and for stimulating their connections. Other authors have since studied this topic (Balkowiec and Katz, 1998; Katz, 2003; Lu, 2003; Kron et al., 2007a,b; Ogier et al., 2013). In particular, Kron et al. (2007a,b) showed the specific involvement of BDNF in both fetal GABAergic inhibitory and postnatal glutamatergic excitatory synaptic transmission in the KF of rats.

This behavior of the BDNF is very likely similar in humans but the related molecular mechanisms of expression and functions are largely unknown. The present study was undertaken to specifically test the hypothesis that the KF nucleus is a target of BDNF signaling to foster a normal breathing pathway. In particular, we supposed that the BDNF can contribute to the inhibitory and stimulatory actions on breathing exerted by the KFN before and after birth, respectively. To approach this issue we applied BDNF immunohistochemistry to serial histological sections of brainstem, particularly of the specific specimen where the KF nucleus (KFN) is located, from subjects who died perinatally of both known and unknown causes. The aims of the research were: firstly to evaluate whether and how the KF neurons display BDNF immunoreactivity; secondly, to investigate whether developmental defects of the KFN, that are not uncommon in sudden perinatal deaths (Lavezzi et al., 2004a,b,c), are associated with alterations of BDNF expression. The study protocol included immunohistochemical detection in those same cases of apoptosis, given the important role of the BDNF also in neuronal survival and/or programmed death occurring during the development of the nervous system (Ricart et al., 2006), and of the Glial Fibrillary Acid Protein (GFAP), a marker of reactive gliosis in neurodegenerative processes (Sofroniew and Vinters, 2010; Robel et al., 2011).

## Methods

A total of 45 brains were studied, from 27 ante-partum stillbirths (25–40 gestational weeks-gw, mean age: 37.5 gw), and 18 infants who survived for periods ranging between 1 h and 6 months (mean age: 3.2 months). All these cases were sent to the “Lino Rossi” Research Center of Milan University according to the application of the Italian law n.31/2006 “*Regulations for Diagnostic Post Mortem Investigation in Victims of sudden Infant Death Syndrome (SIDS) and Unexpected Fetal Death*.” This law decrees that all infants suspected of SIDS who died suddenly in Italian regions within the first year of age, as well as all fetuses who died without any apparent cause (SIUDS: Sudden Intrauterine Unexplained Death Syndrome), must be submitted to a thorough diagnostic post-mortem investigation.

### Consent

Parents of all the victims of the study provided written informed consent to autopsy, and approval was obtained from the Milan University “Lino Rossi” Research Center institutional review board.

**Figure 1:**
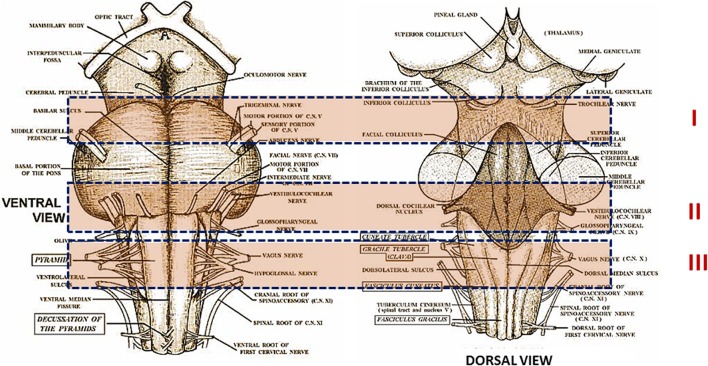
**Schematic representation of the brainstem sampling**. On the left: ventral surface; on the right: dorsal surface. I, ponto-mesencephalic specimen, including the upper third of the pons and the adjacent portion of mesencephalon; II, medullar-pontine specimen, extending from the upper medulla oblongata to the adjacent portion of the caudal pons; III, medulla oblongata specimen, including the obex. Figures are from the “Online Neuroscience Resource”- available at the website: http://www.neuroanatomy.wisc.edu/virtualbrain/BrainStem/24PAG.html.

The victims were subjected to a complete autopsy, including examination of the placental disk, umbilical cord and membranes in fetal deaths. In all cases an in-depth histological examination of the autonomic nervous system was made, according to the protocol provided by the Law 31 (Matturri et al., 2005, 2008). Briefly, examination of the brainstem was performed, after fixation in 10% phosphate-buffered formalin, through the sampling of three specimens, as shown in Figure 1. The first specimen, ponto-mesencephalic, includes the upper third of the pons and the adjacent portion of mesencephalon. The second extends from the upper medulla oblongata to the adjacent portion of the caudal pons. The third specimen takes as reference point the medullary obex, a few mm above and below it.

The three samples were embedded in paraffin and serially cut at intervals of 30 μm. For each level, twelve 5 μm sections were obtained, two of which were routinely stained using alternately hematoxylin-eosin and Klüver-Barrera stains, while three sections were treated for immunohistochemical detection of BDNF, apoptosis and GFAP, respectively. The remaining sections were saved for further investigations and stained as deemed necessary.

The routine histological evaluation of the brainstem was performed on the locus coeruleus and the Kölliker-Fuse nucleus (the focus of this study) in the first specimen; on the parafacial/facial complex, the superior olivary complex and the retrotrapezoid nucleus in the second sample, and on the hypoglossus, the dorsal motor vagal, the ambiguus, the pre-Bötzinger, the inferior olivary, the arcuate nuclei and the solitary tract complex in the third medullary sample.

In 29/45 cases, after the in-depth autoptic examination, the death remained totally unexplained. A diagnosis of “SIUDS” was made for 19 fetuses who died suddenly after the 25th gestational week, and a diagnosis of “SIDS” for 10 infants who died within the first 6 months of life. In the remaining 16 cases, 8 stillbirths, and 8 infants, a precise cause of death was formulated at autopsy. These cases were regarded as “controls.” Table 1 summarizes the subjects analyzed in this study, indicating the sex distribution, range of ages and death diagnoses.

**Table 1:** **Case profiles of the study**.

**Victims**	**Age (range)**	**Sex**		**Death diagnosis**	
		**M**	**F**	**Explained death**	**Unexplained death**
				**Controls**	
				**(n.16)**	**(n.29)**
Fetuses (n.27)	25–40 gw	12	15	Necrotizing chorioamnionitis (n.5) Congenital heart disease (n.3)	SIUDS (n.19)
Newborns (n.18)	1 h–6 m	10	8	Congenital heart disease (n.5) Pneumonia (n.2) Pericarditis (n.1)	SIDS (n.10)

*gw, gestational week; h, hour; m, month; SIDS, Sudden Infant Death Syndrome; SIUDS, Sudden Intrauterine Unexplained Death Syndrome*.

For each case, a complete clinical history, with particular reference to maternal lifestyle (including cigarette smoking, alcohol and drug abuse), was collected. None of the mothers had any significant pathology. Thirteen of the 29 SIUDS/SIDS mothers (45%) were active smokers before and during the pregnancy, smoking >3 cigarettes/day. The remaining 16 mothers (55%) reported no history of cigarette smoking. Three mothers of victims in the control group (19%) had a smoking habit, while 13 mothers (81%) were non-smokers.

#### Immunohistochemical techniques

***BDNF detection***. Sections from paraffin-embedded tissue blocks were stained using commercially supplied rabbit monoclonal antibodies against the brain-derived neurotrophic factor, also known as BDNF [abcam (EPR1292), ab108319]. Slides were boiled for the antigen retrieval in EDTA buffer, using a microwave oven, at 600 W for 3 times at 5 min each, and finally cooled. The antibody was diluted 1:140 in PBS. A standard ABC technique avidin-biotin complex (Vectastain elite ABC KIT, PK-6101) was used with HRP-DAB to visualize and develop the antigen-antibody reaction. All the slides selected for this study were submitted at the same time to the immunohistochemical procedures, with particular attention to the simultaneous incubation in the DAB-peroxidase solution to avoid differences in the immunostaining. Sections were counterstained with Mayer's hematoxylin, than coverslipped.

A set of sections from each group of the study was used as negative control. Precisely, the tissue samples were stained using the same procedure but omitting the primary antibody in order to verify that the immunolabeling was not due to nonspecific labeling by the secondary antibody. In fact, if specific staining occurs in negative control tissues, immunohistochemical results should be considered invalid.

***Quantification of BDNF immunohistochemical expression***. The degree of positive immunoreactivity in the brainstem histological sections was defined for every case by two independent and blinded observers as the number of neurons with unequivocal brown immunostaining, divided by the total number of cells counted in the same area, expressed as percentage (BDNF immunopositivity index: BDNF-Index). BDNF-Index was classified as: “Class 0” for absolutely no staining (negativity); “Class 1” when the index of strong immunopositive neurons was < 10% or even >10% but only of slightly immunopositive cells (weak positivity); “Class 2” with a percentage of intense immunopositive neurons between 10 and 30% (moderate positivity); “Class 3” with a percentage of intensely brown neurons >30% of the counted cells (strong positivity).

All slides were evaluated using a Nikon E600 microscope with Axioplan objectives and identical ND (Neutral Density) filters. Images were acquired at different magnification using a Microlumina Ultra Resolution Scanning Digital Camera.

***Apoptosis detection***. To detect cells undergoing apoptosis, we used the technique of Terminal-Transferase dUTP Nick End labeling technique (TUNEL Apoptag plus peroxidase *in situ* Apoptosis detection kit, S7101, Chemicon). This identifies early nuclear DNA fragmentation by specific binding of terminal deoxynucleotidyl transferase (TdT) to 3′-OH ends of DNA. Sections were pretreated with proteinase k (20 μg/ml) for 15 min. Endogenous hydrogen peroxidase activity was quenched in 3% hydrogen peroxide. After a series of rinsing, nucleotides labeled with digoxigenin were enzymatically added to the DNA by TdT. The incubation was carried out for 60 min the labeled DNA was detected using anti-digoxigenin-peroxidase for 30 min. The chromogen diaminobenzidine tetra hydrochloride (DAB) resulted in a brown reaction product. Incubation without TdT served as the negative control.

***GFAP detection***. To reveal the reactive astrocytes, sections were deparaffinized and washed in PBS. After blocking endogenous peroxidase with 3% H_2_O_2_, the slides were pretreated in a microwave-oven using a citrate solution (pH 6). Then the sections were incubated overnight with primary monoclonal antibody NCL-GFAP-GA5 (anti GFAP, Novocastra, Newcastle Tyne, United Kingdom) at a dilution of 1:300. Immunohistochemical staining was performed with the peroxidase-antiperoxidase method and the avidin-biotin complex technique (ABC Kit, Vectastain, Vector Laboratories Inc., Burlingame, CA, U.S.A.). Diaminobenzidine (DAB, Vector Laboratories Inc., Burlingame, CA, U.S.A.) was used as chromogen substrate and counterstained with light hematoxylin. Negative controls of the same tissue were done using PBS instead of primary antibody.

***GABA (γ-aminobutyric acid) detection***. Immunohistochemistry to identify interneurons, typically inhibitory cells that use the neurotransmitter GABA, was performed by anti-parvalbumin mouse monoclonal IgG antibody (Millipore Chemicon International cat. MAB1572). Sections, after deparaffinizing and rehydrating, were immersed and boiled in a citrate solution pH 6.0 for the antigen retrival with a microwave oven, having first blocked endogenous peroxidase by 3% hydrogen peroxide treatment. Then sections were incubated with the primary antibody overnight (1:1000 dilution) in a wet chamber. Samples were washed with a PBS buffer and processed with a usual avidin-biotin-immunoperoxidase technique and finally counter-stained with Mayer's Hematoxylin and coverslipped. Negative controls were prepared omitting the primary antibody and replacing only with PBS.

#### Statistical analysis

The statistical significance of direct comparisons between the groups of victims was determined using analysis of variance (ANOVA). Statistical calculations were carried out with SPSS statistical software. The differences were statistically significant if *p*-value was <0.05.

## Results

### Morphological and immunohistochemical examination of the KFN

#### Control group

**Figure 2:**
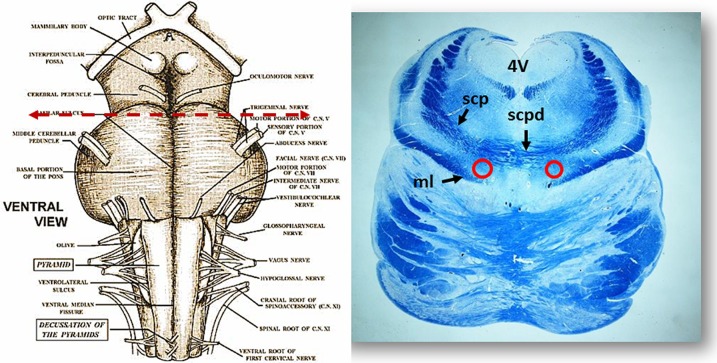
**On the left side the ventral brainstem surface scheme with the indication of the level of the histological section in the rostral pons, visible to the right**. Circles show the bilateral localization of the Kölliker-Fuse nucleus. Klüver Barrera staining. ml, medial lemniscus; scp, superior cerebellar peduncle; scpd, decussation of the superior cerebellar peduncles; 4V, fourth ventricle.

**Figure 3:**
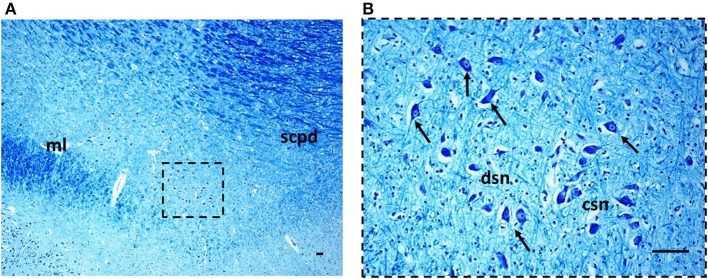
**The Kölliker-Fuse nucleus in a histological section of rostral pons of a control newborn (3 month-old)**. The boxed area in **(A)** is represented at higher magnification in **(B)**. At this magnification it's possible to recognize the large Kölliker-Fuse neurons with distinct nucleus and evident nucleolus (arrows indicate some of these neurons), intermixed to smaller cells (interneurons and astrocytes). The large neurons can be subdivided in two groups: a compact subnucleus and a dissipatus subnucleus. Klüver Barrera staining. Scale bar = 10 μm. csn, compact subnucleus; dsn, dissipatus subnucleus; ml, medial lemniscus; scpd, decussation of the superior cerebellar peduncles.

***Morphology***. We firstly examined the KFN in serial histological sections from the first brainstem sample (Figure 1) of all 16 subjects who died of known causes, with the aim of delineating the normal features of this nucleus in human perinatal life. This examination reaffirmed in more detail our previous reports on this topic (Lavezzi et al., 2004a,b), establishing that the KFN, already well developed at 25 gestational weeks, extends longitudinally from the rostral pons to the lower portion of the mesencephalon, up to the level just where the caudal pole of the red nucleus appears. Its cytoarchitecture is well visible in the more cranial transverse sections of the pons, namely those bordering the caudal mesencephalon, identifiable by the presence of the superior cerebellar peduncle decussation. Figure 2 represents a transverse cranial section of rostral pons showing the location of the KFN. The KFN appears as a group of large neurons, located between the peduncle crossing and the medial lemniscus. These neurons show a distinct, eccentric nucleus with an evident nucleolus, and abundant cytoplasm with Nissl substance located at the cell periphery. On the basis of the neuronal arrangement, it is possible to define two KF subnuclei: the “compactus subnucleus,” consisting of a cluster of a few large neurons, and the adjacent “dissipatus subnucleus” with scattered neurons. Intermixed with these large neurons, smaller cells (interneurons and astrocytes) are visible (Figure 3). Interneurons in particular, in contrast to the typical morphological features of the large KF neurons, had, beyond the lower cell body size, wrinkled nuclei, indistinct nucleoli, inconspicuous Nissl bodies and small extension of the dendritic arbor. In addition, their presence has been confirmed by the specific immonohistochemical method using the anti-parvalbumin antibody as marker for inhibitory GABAergic interneurons.

**Figure 4:**
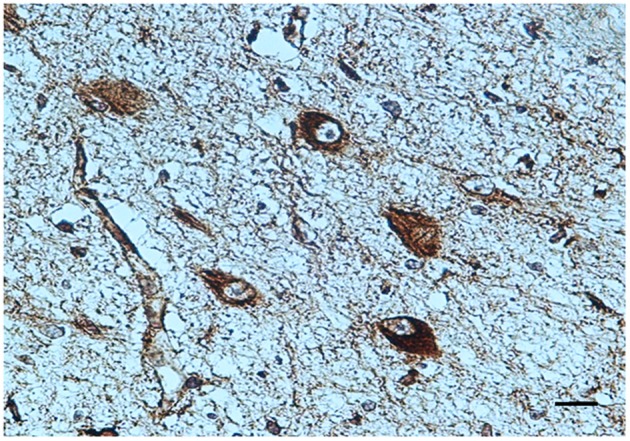
**BDNF immunopositivity of several neurons of the Kölliker-Fuse nucleus in a fetus of the control group (34 gestational weeks)**. BDNF immunostaining. Scale bar = 10 μm.

***BDNF immunohistochemistry***. A noteworthy observation was that the KF neurons display BDNF immunoreactivity prevalently in fetal life (Figure 4). An intense expression with dark brown staining in the cytoplasm of neurons (“Class 3” of BDNF-Index) was seen in all the 8 fetuses and in 1 newborn, who died in the first hours of life. Only in a 3 month-old victim a weak immunopositivity (“Class 1” of BDNF-Index) of the KF neurons was observed.

**Figure 5:**
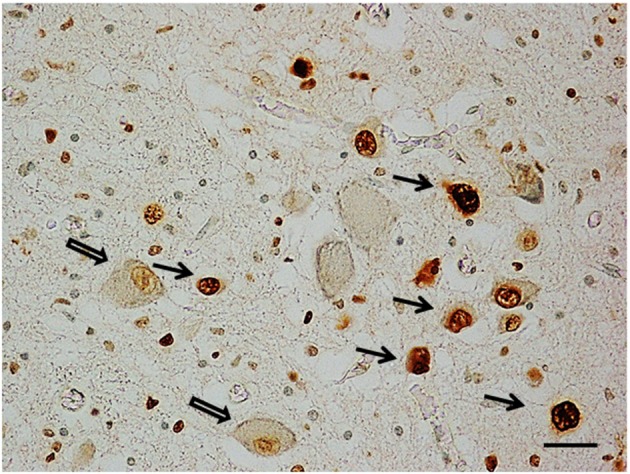
**Apoptotic interneurons in the Kölliker-Fuse nucleus in a newborn of the control group (2 month-old)**. Full arrows indicate immunopositive interneurons; empty arrows indicate some of the immunonegative large neurons. TUNEL metod immunostaining. Scale bar = 10 μm.

***Apoptosis immunohistochemistry***. Significant apoptosis, after application of the TUNEL method, was absent in the KFN of the fetal control group. Programmed cell death was, instead, confined to several interneurons in the KF area in 7 of the 8 control infants (Figure 5).

***GFAP immunohistochemistry***. Rare astrocytes not expressing detectable levels of GFAP with none sign of immunopositive reactive astrogliosis were found in the KFN area of all the control cases.

#### SIUDS/SIDS group

**Figure 6:**
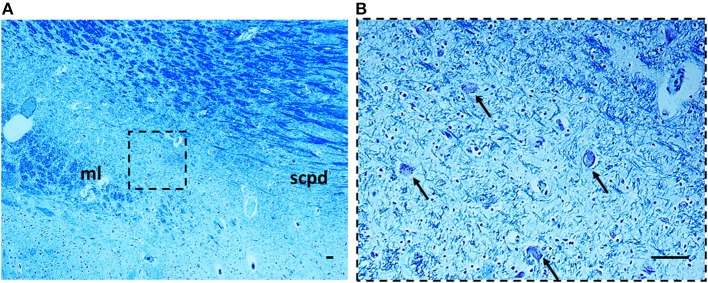
**Hypoplasia of the Kölliker-Fuse nucleus in a SIUDS case (39 gestational weeks)**. The boxed area in **(A)** is represented at higher magnification in **(B)**. At this magnification only rare suffering Kölliker-Fuse neurons are visible (see arrows). Klüver Barrera staining. Scale bar = 10 μm. ml, medial lemniscus; scpd, decussation of the superior cerebellar peduncles.

***Morphology***. In 14 subjects who died suddenly (7 SIUDS and 7 SIDS), the KF structure did not differ from those of age-matched controls. However, a decreased number of KF neurons (hypoplasia) was observed in 9 late fetal deaths aged from 38 to 40 gestational weeks and in 3 newborns who died within the first day of life (Figure 6). In 3 stillbirths (28–32 gestational week-olds), the KFN was not detectable, unlike to the age-matched control fetuses in which the KF is already well-developed, thus allowing a diagnosis of “agenesis” of this nucleus.

**Figure 7:**
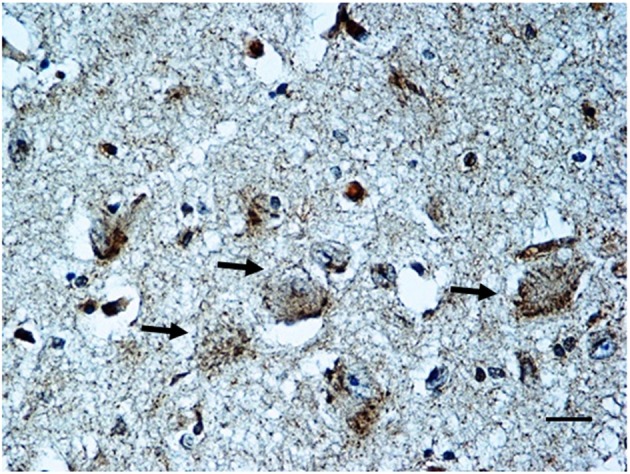
**BDNF weak immunopositivity of the Kölliker-Fuse nucleus in a SIUDS case (35 gestational weeks)**. Arrows indicate the large Kölliker-Fuse neurons. BDNF immunostaining. Scale bar = 10 μm.

***BDNF immunohistochemistry***. Results consistent with the control group observations were found in 17 cases (positive BDNF immunoexpression in 10 SIUDS and negative immunoexpression in 7 SIDS). An irregular BDNF expression was observed in the remaining 12 cases: negativity (Class 0 of BDNF-Index) was found in the three fetuses with KFN agenesis and in 4 sudden deaths with hypoplasia; very weak to moderate immunopositivity (“Class 1” and “Class 2” of BDNF-Index), only distributed at the cell periphery, was highlighted in 2 SIUDS cases (Figure 7) and in 3 newborns died in the first hours of life with KFN hypoplasia. Collectively, a significantly greater proportion of BDNF signaling dysfunctions were observed in SIUDS/SIDS victims compared with controls (41 vs. 12%, *p* < 0.05).

***Apoptosis immunohistochemistry***. Contrary to what was observed in controls, positive TUNEL staining was detected in the KF interneurons of 8 SIUDS cases, and negative apoptotic signals in 4 SIDS. Frequently these results were associated to negative/weak BDNF expression and KFN hypoplasia.

**Figure 8:**
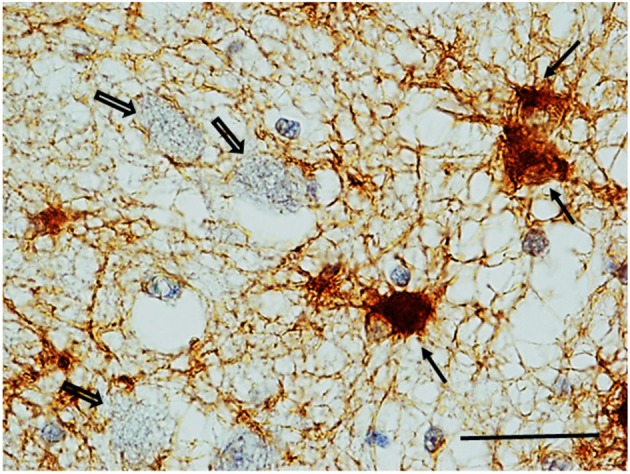
**Reactive astrocytes (see full arrows) in the Kölliker-Fuse area in a SIDS case (2 month-old)**. These cells are characterized by high-level expression of GFAP immunoreactivity in both hypertrophic cell bodies and processes that appear striking increased in number, length and thickness. Empty arrows indicate some of the large GFAP-immunonegative large neurons of the Kölliker-Fuse nucleus. GFAP immunohistaining. Scale bar = 10 μm.

***GFAP Immunohistochemistry***. Numerous reactive astrocytes, characterized by high-level expression of GFAP immunoreactivity in spongiform hypertrophic cell bodies and striking increase in the number, length and thickness of GFAP-positive processes, were found nearby the large neurons of the KFN in about 40% of both SIUDS and SIDS victims (8/19 and 4/10, respectively) (Figure 8).

In Table 2, all the results related to the KFN are reported. Overall, we found one or more combinations of KFN pathological findings (i.e., hypoplasia, gliosis, irregular manifestation of the BDNF and apoptosis) in 19 victims of sudden death (14 SIUDS and 5 SIDS) and only in 1 control group case. Thus, a significantly greater proportion of neurological alterations of the KFN was observed in victims of sudden death as compared with controls (*p* < 0.01).

**Table 2:** **Morphological and immunohistochemical findings related to KFN in SIUDS/SIDS and Controls**.

**Victims**	**Morphology**			**BDNF immunohistochemistry Index:**			**TUNEL method**		**GFAP method**	
	**Normal**	**Hypoplasia**	**Agenesis**				**Positive**	**Negative**	**Positive**	**Negative**
				**Class 3**	**Class 1/2**	**Class 0**				
				**Strong**	**Weak/Moderate**	**Negative**				
**CONTROLS**										
Fetuses (n.8)	8	–	–	8	–	–	–	8	–	8
Infants (n.8)	8	–	–	1^a^	1	6	7^b^	1	–	8
**SUDDEN DEATH**										
SIUDS (n.19)	7	9	3	8	2(1^c^)^*^	7^c^^**^	8^b^^**^	–	8^**^	11
SIDS (n.10)	7	3^a^	–	–	3^a^,^c^	7	6^b^	4	4	6

*SIDS, Sudden Infant Death Syndrome; SIUDS, Sudden Intrauterine Unexplained Death Syndrome*.

a *Died in the first hours of life*.

b *Confined to interneurons*.

c *With KFN hypodevelopment*.

*Statistically significant differences SIUDS and SIDS vs. corresponding controls (fetuses and newborns, respectively)*:

* *p < 0.05*;

** *p < 0.01*.

## Other brainstem pathological results

A subset of SIUDS (11/19) cases showed hypoplasia of the arcuate and the pre-Bötzinger nuclei in the medulla oblongata, and of the facial parafacial complex in the caudal pons. In 4 cases these alterations were concomitant with KFN hypoplasia. In the SIDS group the most frequent alteration was hypoplasia of the arcuate nucleus (6/10). Also in 25% of the control cases (4/16), both fetal and infant deaths, hypoplasia of the arcuate nucleus was detected.

In the majority of cases (both control and SIUDS/SIDS groups), regardless of the expression pattern in the KFN, the BDNF was also densely expressed in the ventrolateral subnucleus of the solitary tract complex, in the pre-Bötzinger nucleus in the medulla oblongata, and in the retrotrapezoid nucleus in the caudal pons.

### Correlation of KFN findings with smoke exposure

On the whole, the morphological and immunohistochemical alterations of the KFN resulted significantly related to maternal smoking (*p* < 0.01). In fact, in 10 of the 13 victims of sudden death with a smoker mother (77%) one or more developmental defects of the KFN were present. Similarly, in the control group, 2 of the 3 newborns (67%) showing anomalous BDNF positive immunoexpression, had a smoking mother, confirming the role of smoke absorption in the neuropathogenetic manifestation of this growth factor.

## Discussion

From the experimental reports in literature there is clear evidence that the neurotrophin BDNF is important for the maturation of the respiratory network during the prenatal period and for the primary generation of the respiratory rhythm at birth. In addition, BDNF has an important role in the stabilization of central breathing that occurs after birth (Balkowiec and Katz, 1998; Katz, 2003; Lu, 2003; Ogier et al., 2013).

In this study we examined the expression of BDNF in humans, mainly in the KFN, an essential component of the respiratory network with important implications before and after birth. Our research was performed on a wide set of fetuses and newborns who died of both known (controls) and unknown causes (SIUDS/SIDS).

Only limited data are available at this moment regarding the localization and the cytoarchitecture of the human KFN. In this study we have shown, validating our previous reports (Lavezzi et al., 2004a,c), that the KFN is well analyzable in histological sections from the dorsolateral area of the cranial pons, ventral to the superior cerebellar peduncle decussation, Our data are supported by the study of Pattinson et al. (2009). These authors, through combined functional and structural magnetic resonance imaging techniques applied to brainstem of human volunteers, observed, after chemical stimulation of breathing, an intense activation in a brain area of the rostral dorsal pons corresponding to the KFN localization highlighted by us. In the selected histological sections of control cases we first defined the cytoarchitecture of this nucleus, consisting of a small population of large neurons intermixed with smaller interneurons and glial cells. Then, we found developmental changes in the cytoarchitectural organization and in the immunoexpression of BDNF in the KFN, particularly in sudden fetal deaths (SIUDS). This was illustrated in particular by the prevalent negativity of BDNF signals, unlike the intense expression found in age-matched fetal controls, that coincides with the peculiar BDNF function as breathing-inhibitor shown in experimental studies in prenatal life (Kron et al., 2007b). The disappearance of positivity observed after birth, above all in control cases, has been interpreted, on the contrary, as a necessary step to allow for breathing to start. The BDNF immunopositivity, even if weak, observed in the KFN of several SIDS cases can therefore indicate a continuation of the prenatal inhibitory activity after birth, leading to severe respiratory deficits and consequently death.

A remarkable observation was that BDNF deficiency in the KF neurons of SIUDS and its expression in SIDS victims were frequently associated with a delayed morphological maturation of this nucleus (KFN hypoplasia), thus demonstrating that alterations of BDNF expression can derange the KF maturation.

Therefore, the data obtained from this study essentially indicate that: (1) BDNF has direct effects on breathing inhibition in fetal life; (2) BDNF is required for normal KFN development in intrauterine life but is not involved in starting the breathing and the modulation promoted by the KFN after delivery. Indeed, BDNF expression in newborns seems to hinder the ventilatory activity.

Our considerations are, in general, in agreement with the report by Tang et al. (2012), providing evidence of an abnormal expression of BDNF in respiratory-related brainstem structures (although the KFN was not included in their observations) in SIDS as compared to non-SIDS infants.

The results highlighted by the application of TUNEL immunohistochemistry are also interesting. We observed, in fact, the presence of apoptotic interneurons in the KFN of newborns, mainly of the control group.

Interneurons are typically small cells distributed in the central nervous system (CNS), that form short-distance connections with neighboring large neurons through the use of the neurotransmitter GABA to provide a synaptic inhibitory control (Sato et al., 2014). During gestation the activity of the respiratory muscles is disabled by the growing number of inhibitory interneurons, densely distributed above all in the KFN area. At physiological delivery, these interneurons are generally stressed, consuming plenty of oxygen and glucose (Funk et al., 2008). They are then eliminated by apoptosis, as we have tested in the KFN of control subjects, thus allowing the breathing to start (Morpurgo et al., 2004). The high percentage of SIDS cases without apoptotic signals in the KFN observed in this study could be attributable to the persistence, after birth, of active interneurons that preserve their inhibitory effect on the ventilation, thus leading to a fatal conclusion.

The reactive GFAP-immunopositive astrocytes we found around the KF neurons in SIUDS/SIDS victims should also be underlined. Astrocytes are the major glial cell population within the CNS. They play important physiological roles in brain functions through the release of several neurotrophic factors that represent the primary event in the maintenance of CNS homeostasis, providing support and protection to neurons (Hughes et al., 2010; Jones and Bouvier, 2014). In addition, astrocytes have the ability to rapidly react to various noxious neuronal insults, leading to vigorous astrogliosis (Gourine et al., 2010; Robel et al., 2011). Subsequently, after severe activation, astrocytes secrete neurotoxic substances and express an enhanced level of glial fibrillary acidic protein (GFAP), which is mostly considered to be a marker of hypoxic states (Chekhonin et al., 2003). Hypoxia induces the proliferation of activated astrocytes particularly in specific brain regions that play an important role in the physiological control of breathing, such as the KFN (Becker and Takashima, 1985; Norenberg, 1994).

In our study an important risk factor responsible for hypoxic conditions has been identified in cigarette smoke, given the high incidence of smoker mothers. In cases of cigarette smoke absorption in pregnancy, nicotine and carbon monoxide easily cross the placental barrier and bind to the fetal hemoglobin. The resulting carboxyhemoglobin is not able to release oxygen, consequently inducing alterations of the physiological development of those organs and tissues most susceptible to hypoxic damage, including the brain (Lichtensteiger et al., 1988; Levin and Slotkin, 1998; Gressens et al., 2003). Besides, nicotine is one of the few lipid-soluble substances that can cross the blood-brain barrier and act directly by inducing specific molecular alterations in DNA, RNA, and proteins of the nervous cells (Gospe et al., 1996).

Smoking may also be responsible for aberrant BDNF behaviors. In fact, we frequently observed an altered expression of BDNF in SIUDS/SIDS subjects with smoker mothers. In support of this hypothesis is the demonstration that a multitude of stimuli, including nicotine absorption, alter *BDNF* gene expression, by modifying *BDNF* mRNA in specific neuronal structures (Lindholm et al., 1994).

It is important to point out several limitations of this study. Firstly, the relatively small number of cases of SIUDS and particularly of SIDS and controls, that allows us to formulate hypotheses but not gain confirmation. Secondly, failure to make a specific examination of the tropomyosin-related kinase B (TrkB) receptor, a protein with high affinity toward several neurotrophins, that interacts in particular with BDNF to mediate neuronal growth during CNS development (Soppet et al., 1991; Aoki et al., 2000; Tang et al., 2010). Such research is now in progress in our Research Center, however, as we are planning to examine the behavior of this specific receptor in a wider set of perinatal deaths.

In conclusion, we can state that BDNF appears to be required for the development of the KFN in human fetal life and for its inhibitory function on the central respiratory output occurring *in utero*. Interestingly, the BDNF role in the breathing mechanism is different in fetal and neonatal life. In fact, in contrast with the indispensable high BDNF expression in KF neurons *in utero*, its manifestation in newborns results associated to severe impairment of the respiratory activity.

In any case, the altered behavior of the BDNF system observed in SIUDS/SIDS victims may hinder the KFN function, compromising the respiratory control in both fetal and postnatal life.

Our findings differ from the reports in literature related to experimental studies, showing BDNF signaling in KF neurons also after birth. It should be noted, however, that this different behavior in man only affects the KFN. In fact, BDNF was expressed in other respiratory structures (in particular the solitary tract complex, the pre-Bötzinger and the retrotrapezoid nuclei) in all stages of life. These observations emphasize the need to intensify the studies on BDNF expression in the human brainstem.

Finally, we point out that BDNF, as a neuromodulator in human KF, may have a physiological importance and clinical relevance in a wide range of human developmental disorders of the breathing. A dysregulation of BDNF expression has been linked with Rett's syndrome (RTT), for example, a childhood neurological disease associated with a mutation in the gene *MeCP2* and serious impairment of the ventilatory activity (Rett, 1986; Katz et al., 2009). Mironov et al. (2009) demonstrated, in a mouse model of RTT, that BDNF deficits are most prominent in neuronal structures of the brainstem deputed to autonomic and respiratory control. Importantly, Wang et al. (2006) revealed, in M*eCP2* null mice, significant decreased levels of BDNF expression in the nucleus of the solitary tract, a nucleus proposed as source for BDNF release in the KFN.

In the idiopathic congenital central hypoventilation syndrome (CCHS), a rare disorder characterized by an abnormal control of respiration despite the absence of neuromuscular or lung disease or an identifiable brainstem lesion (Weese-Mayer et al., 1992), several authors have shown an important contribution of BDNF system dysfunction to the pathophysiological mechanism leading to this specific respiratory deficiency. Chiaretti et al. (2005), in particular, found a reduction of BDNF in the cerebrospinal fluid of patients affected by Ondine's curse compared with the mean level in the control group. Weese-Mayer (Weese-Mayer et al., 2002) highlighted a mutation of the *BDNF* gene, although not very frequent, in children with CCHS, supporting the relevance of this identified mutation in the respiratory control.

A further interesting point to verify will be whether the link between BDNF deregulations and breathing activity perturbations highlighted in these pathologies originates from an impaired synaptic transmission and developmental alterations of the KFN.

## Author contributions

Anna M. Lavezzi planned the study, analyzed the data and wrote the manuscript with collaborative input and extensive discussion with Luigi Matturri. Melissa F. Corna carried out the immunohistochemical and the histochemical study and participated in the evaluation of the results. All Authors read and approved the final manuscript.

### Conflict of interest statement

The Review Editor Maria Elena Ferrero declares that, despite being affiliated to the same institution as authors Anna M. Lavezzi, Melissa F. Corna and Luigi Matturri, the review process was handled objectively and no conflict of interest exists. The authors declare that the research was conducted in the absence of any commercial or financial relationships that could be construed as a potential conflict of interest.
